# Association of the *RBFOX1* rs6500744 Polymorphism with Periodontal Disease Severity in Adults with Obesity: A Case–Control Study

**DOI:** 10.3390/ijms27114867

**Published:** 2026-05-28

**Authors:** Felicia Gabriela Beresescu, Daniela-Tatiana Sala, Alina Ormenisan, Andrea Bors, Melinda Székely, Liana Beresescu, Adriana-Stela Crisan

**Affiliations:** 1 Faculty of Dental Medicine, George Emil Palade University of Medicine, Pharmacy, Science and Technology of Targu Mureș, 540142 Targu Mureș, Romania; 2 2nd Department of Surgery, George Emil Palade University of Medicine, Pharmacy, Science and Technology of Targu Mureș, 540142 Targu Mureș, Romania; 3 2nd Clinic of Surgery, Mureș County Emergency Clinical Hospital, 540136 Targu Mureș, Romania; 4 Genetics Department, George Emil Palade University of Medicine, Pharmacy, Science and Technology of Targu Mureș, 540136 Targu Mureș, Romania; 5 Genetics Laboratory, Center for Advanced Medical and Pharmaceutical Research, George Emil Palade University of Medicine, Pharmacy, Science and Technology of Targu Mureș, 540139 Targu Mureș, Romania

**Keywords:** periodontitis, obesity, *RBFOX1*, genetic polymorphism, multimorbidity, biomarker

## Abstract

Obesity and periodontitis are chronic diseases that share inflammatory and metabolic pathways. The *RBFOX1* gene was selected as an exploratory candidate gene because it encodes an RNA-binding splicing regulator, has been implicated in obesity-related phenotypes, and has been reported in candidate-gene analyses of periodontitis/metabolic traits. This study investigated the association between obesity, periodontal disease severity, and *RBFOX1* rs6500744 polymorphism. This case–control study enrolled 106 adults: 53 with obesity (BMI ≥ 30 kg/m^2^) and 53 normoweight controls. Clinical and radiographic periodontal assessments determined disease severity, complexity, staging, and grading. Genotyping of the *RBFOX1* rs6500744 polymorphism (CC, CT, TT) was performed using the TaqMan^®^ SNP Genotyping Assay and the 7500 Fast Dx Real-Time PCR system. Multivariable models adjusted for age, sex, smoking status, and clinically confirmed type 2 diabetes. Participants with obesity showed higher levels of periodontal disease than normoweight controls, indicated by greater pocket depths, attachment loss, and bone loss. Advanced stage (Stage III/IV; *p* = 5.35 × 10^−6^) and Grade C periodontitis (*p* = 2.08 × 10^−7^) were significantly more frequent in the obese group. The rs6500744 T allele was more common among individuals with obesity (46.2% vs. 30.2%; OR 1.99, *p* = 0.023). Periodontal damage appeared to increase progressively from CC to CT to TT genotype, but genotype-stratified estimates, particularly for TT homozygotes, were interpreted cautiously because of the small subgroup size and multiple testing. Both obesity and the number of T alleles were associated with increased periodontal severity in adjusted statistical models. VIFs were low, residual diagnostics did not indicate major assumption violations, smoking-stratified sensitivity analyses were directionally consistent, and the obesity × T-allele interaction was not statistically significant. Obesity is associated with more severe and extensive periodontal disease in this exploratory case–control cohort; however, residual confounding from significant smoking imbalances between groups cannot be excluded. The *RBFOX1* rs6500744 T allele may mark increased susceptibility to periodontal tissue destruction, but the findings do not establish causality or clinical prognostic utility and require longitudinal validation in larger, ancestry-controlled populations.

## 1. Introduction

Obesity and periodontitis are chronic, highly prevalent non-communicable diseases and pose major global public health challenges. Obesity rates have risen sharply in recent decades. Severe periodontitis affected more than one billion people worldwide as of 2021 [1,2,3,4]. Both conditions reduce quality of life, increase healthcare utilization, and often co-occur with other systemic inflammatory and metabolic conditions [5]. Periodontitis is characterized by dysbiotic biofilm-induced inflammation and destruction of tooth-supporting tissues and is now understood within a wider systemic context. Current classification structures encourage the integration of systemic susceptibility and biologic risk into periodontal assessment [6,7,8,9]. Importantly, periodontal tissue destruction is not driven solely by microbial accumulation or by host genetics in isolation; it reflects dynamic host–microbial interactions in which a dysbiotic biofilm amplifies immune-inflammatory, oxidative, and bone-resorptive pathways [6,10,11,12,13,14,15].

Emerging epidemiological evidence indicates a strong association between excess adiposity, both overall and central adiposity, and worsening periodontal status [6]. Recent studies and meta-analyses show that overweight and obese individuals are more likely to develop severe periodontitis [9,10]. This relationship may be influenced by overlapping pathways, such as chronic low-grade systemic inflammation, oxidative stress, insulin resistance, immune dysregulation, and changes in the secretion of adipose-derived mediators [5,11]. As a result, the clinical perspective now views obesity, dysglycemia, and periodontitis through a multimorbidity framework [5,11]. In this context, severe periodontal destruction in patients with obesity may signal a wider systemic metabolic dysfunction rather than a problem limited to oral health [5,11].

Microbial, behavioral, and systemic factors are central to periodontitis, but they cannot fully explain interindividual variation in periodontal tissue destruction [13]. Heritability analyses suggest that genetic factors account for a meaningful proportion of variance in periodontitis susceptibility [14], although the disease’s genetic architecture is complex, polygenic, and heterogeneous [15]. Genome-wide studies have yielded inconsistent results across populations [16,17], supporting cautious examination of biologically plausible candidate variants that may connect metabolic disease with periodontal risk.

*RBFOX1* (formerly *A2BP1*) was selected a priori as the candidate gene for this study for three reasons. *RBFOX1* encodes an evolutionarily conserved RNA-binding protein involved in tissue-specific alternative splicing [18]. Genetic variation in *A2BP1/RBFOX1* has been evaluated in relation to obesity-related phenotypes, including adiposity [19,20]. A prior candidate-gene study reported *RBFOX1* involvement in the interaction between periodontitis and metabolic traits [21]. The rs6500744 polymorphism was therefore selected as an exploratory marker within a metabolically relevant locus with an available validated TaqMan assay, rather than as an established causal or prognostic variant. Experimental evidence indicating that Rbfox family proteins participate in pancreatic beta-cell function and survival provides additional, indirect biological plausibility [22], but functional periodontal evidence for rs6500744 remains unavailable.

This exploratory study aimed to assess the association between obesity and periodontal disease severity and to examine whether the *RBFOX1* rs6500744 polymorphism relates to obesity status and periodontal phenotype in the study population. The analysis was not designed to establish causality or to validate a prognostic genetic model.

## 2. Results

The study population comprised 106 participants, evenly distributed between normoweight controls (n = 53) and obese cases (n = 53). The groups were well matched regarding age (44.55 ± 7.51 vs. 46.28 ± 6.87 years, *p* = 0.217) and sex distribution (women: 43.4% vs. 56.6%, *p* = 0.244). However, smoking habits differed significantly between the cohorts. Current smokers were more common in the obese group (37.7% vs. 11.3%), while never-smokers were more frequent among controls (79.2% vs. 43.4%) (*p* = 6.24 × 10^−4^). Consequently, cumulative smoking exposure (pack-years) was significantly higher in obese subjects (7.03 ± 6.87 vs. 1.85 ± 3.87, *p* = 5.77 × 10^−6^). Type 2 diabetes was clinically confirmed from medical records or current antidiabetic treatment; its prevalence was higher in the obese group than in controls (18.9% vs. 5.7%, *p* = 0.072). HbA1c values were available for all participants and were higher in the obese group (6.34 ± 0.53% vs. 5.46 ± 0.37%, *p* = 1.46 × 10^−16^). As anticipated, the obese cohort exhibited significantly higher body mass index (BMI) (35.64 ± 2.82 vs. 23.00 ± 1.52 kg/m^2^) and waist circumference (WC) (114.27 ± 6.16 vs. 89.86 ± 6.44 cm) (BMI: *p* = 2.71 × 10^−51^; WC: *p* = 2.57 × 10^−37^).

**Figure 1:**
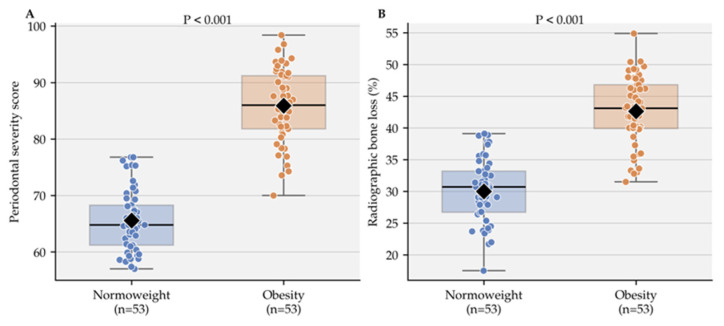
Group difference in periodontal burden. (**A**) Periodontal severity score by study group. (**B**) Radiographic periodontal bone loss (%) by study group.

Periodontal Burden and Classification. Periodontal involvement was consistently and significantly more severe among participants with obesity. Compared with controls, the obese group retained fewer teeth (26.32 ± 0.94 vs. 27.42 ± 0.77, *p* = 2.02 × 10^−9^), lost more teeth specifically due to periodontitis (1.68 ± 0.94 vs. 0.58 ± 0.77, *p* = 2.02 × 10^−9^), and had a greater number of teeth exhibiting clinical attachment loss (CAL) ≥3 mm (14.57 ± 2.82 vs. 10.70 ± 2.63, *p* = 5.77 × 10^−11^). Furthermore, all evaluated clinical parameters were significantly elevated in the obese group, including mean probing pocket depth (PPD) (4.85 ± 0.20 vs. 4.18 ± 0.22 mm), maximum PPD (6.16 ± 0.26 vs. 5.33 ± 0.30 mm), mean CAL (4.00 ± 0.22 vs. 3.33 ± 0.24 mm), and maximum CAL (5.37 ± 0.35 vs. 4.60 ± 0.37 mm) (*p* ≤ 6.56 × 10^−19^). Inflammatory and structural markers followed this pattern, with significantly higher bleeding on probing (BOP) (67.54 ± 4.02% vs. 55.72 ± 4.81%), plaque index, gingival index, radiographic periodontal bone loss (PBL) (42.64 ± 5.37% vs. 30.00 ± 4.94%), PBL/age ratio, furcation involvement, tooth mobility, and overall composite periodontal severity score (85.91 ± 6.43 vs. 65.57 ± 5.37) in the obese cohort (*p* ≤ 1.03 × 10^−12^) (Figure 1).

**Figure 2:**
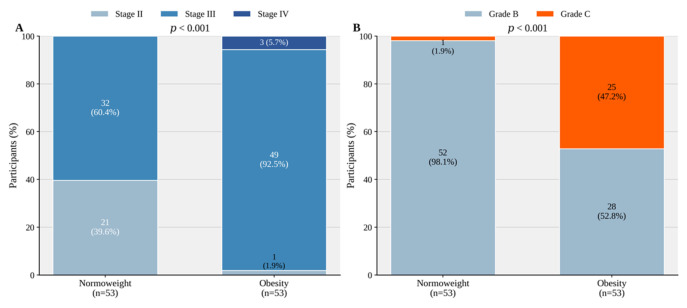
Periodontal stage and grade distributions by study group (normoweight controls n = 53; obese participants n = 53). (**A**) shows the distribution of stage II, III, and IV periodontitis among normoweight and obese participants. (**B**) shows the distribution of grade B and grade C periodontitis by group.

Categorical periodontal classifications mirrored these continuous clinical findings. Generalized disease affected 100% of the obese subjects compared with 83.0% of controls (*p* = 0.003). Advanced Stage III/IV periodontitis was diagnosed in 98.1% of the obese group versus 60.4% of the normoweight group, while Stage II disease was confined to controls (39.6% vs. 1.9%, *p* = 4.22 × 10^−6^). Similarly, rapidly progressing Grade C periodontitis was overrepresented in the obese cohort (47.2% vs. 1.9%, *p* = 2.08 × 10^−7^) (Figure 2).

**Figure 3:**
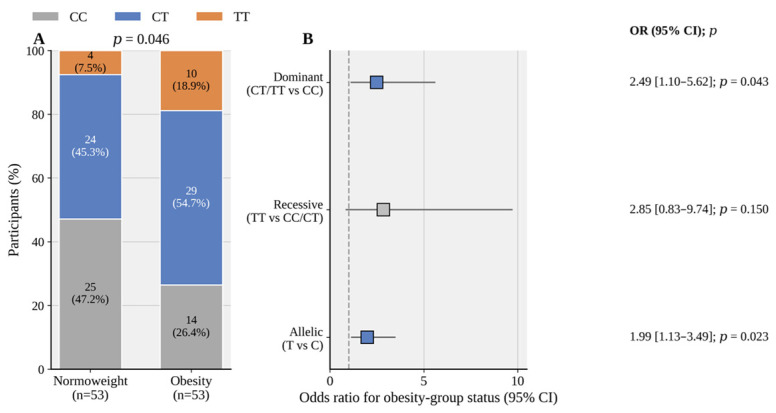
Association between the *RBFOX1* rs6500744 SNP and obesity-group status. (**A**) shows the distribution of rs6500744 genotypes among normoweight controls (n = 53; CC n = 25, CT n = 24, TT n = 4) and obese participants (n = 53; CC n = 14, CT n = 29, TT n = 10). (**B**) shows odds ratios with 95% confidence intervals for obesity-group status under dominant, recessive, and allelic genetic models.

Genotype Distribution of the *RBFOX1* rs6500744 Polymorphism. The distribution of rs6500744 genotypes differed significantly between the two study groups (*p* = 0.046). In the control group (n = 53), the genotype counts and frequencies were CC 25 (47.2%), CT 24 (45.3%), and TT 4 (7.5%), whereas in the obese group (n = 53), the distribution shifted toward the T allele: CC 14 (26.4%), CT 29 (54.7%), and TT 10 (18.9%). The observed genotype frequencies were consistent with Hardy–Weinberg equilibrium by chi-square goodness-of-fit testing in the control group (*p* = 0.588), which was treated as the primary case–control quality-control check. Overall-sample and obese-subgroup HWE results are reported descriptively only (overall *p* = 0.544; obese subgroup *p* = 0.464), because deviation in cases could reflect association rather than genotyping error. Under a dominant genetic model, carriers of at least one T allele (CT + TT) were significantly more prevalent in the obese group (73.6% vs. 52.8%; OR = 2.49, 95% CI 1.10–5.62, *p* = 0.043). At the allelic level, the T allele frequency was 46.2% in obese cases compared with 30.2% in controls (OR = 1.99, 95% CI 1.13–3.49, *p* = 0.023). Although the recessive model (TT vs. CC + CT) showed a similar directional trend, the association did not achieve statistical significance (18.9% vs. 7.5%; OR = 2.85, 95% CI 0.83–9.74, *p* = 0.150) and was interpreted cautiously in view of the small TT subgroup and multiple comparisons (Figure 3).

**Figure 4:**
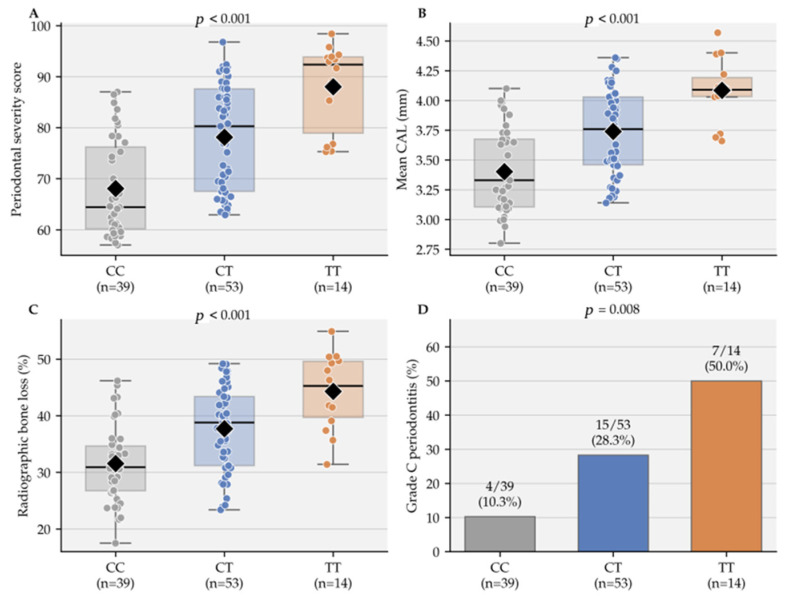
Periodontal severity increases across rs6500744 genotypes (CC n = 39, CT n = 53, TT n = 14). (**A**) shows the composite periodontal severity score by genotype. (**B**) shows the mean clinical attachment loss (CAL). (**C**) shows radiographic periodontal bone loss (PBL). (**D**) shows the proportion of participants with Grade C periodontitis in each genotype group.

Relationship Between Genotype and Periodontal Severity. Across the whole cohort (CC n = 39, CT n = 53, TT n = 14), periodontal severity increased progressively from CC to CT to TT genotypes. This genotype-related gradient should be regarded as an exploratory allele–dose pattern rather than evidence of causality. The statistical certainty of these genotype-stratified estimates, particularly for the small TT homozygote subgroup, is limited and the results were interpreted with caution because multiple periodontal comparisons were performed (Figure 4). The composite periodontal severity score increased in a stepwise manner from 68.08 ± 9.47 in CC carriers to 78.13 ± 10.35 in CT carriers, peaking at 88.02 ± 8.49 in TT carriers (*p* = 2.08 × 10^−9^). This gradient was consistent across specific clinical metrics, including mean PPD (4.29 ± 0.36 to 4.58 ± 0.37 to 4.85 ± 0.25 mm), maximum PPD, mean CAL (3.40 ± 0.35 to 3.74 ± 0.35 to 4.09 ± 0.27 mm), and maximum CAL (*p* ≤ 1.49 × 10^−6^). Inflammatory and radiographic parameters followed this pattern: BOP increased from 57.12 ± 7.04% (CC) to 63.26 ± 6.04% (CT) to 68.00 ± 5.99% (TT) (*p* = 1.70 × 10^−7^), and radiographic PBL increased from 31.57 ± 6.76% to 37.71 ± 7.34% to 44.30 ± 6.73%, respectively (*p* = 1.09 × 10^−7^). Similar progressive worsening across genotypes was noted for plaque index, gingival index, furcation involvement, tooth mobility, and teeth lost due to periodontitis (*p* ≤ 3.26 × 10^−5^). Considering these continuous clinical measures, the prevalence of Grade C periodontitis increased according to genotype: 10.3% in CC, 28.3% in CT, and 50.0% in TT carriers (*p* = 0.008). Genotype was also associated with disease staging (*p* = 2.05 × 10^−6^). Stage IV disease was observed only among TT carriers, and all Stage IV cases occurred in obese subjects.

**Figure 5:**
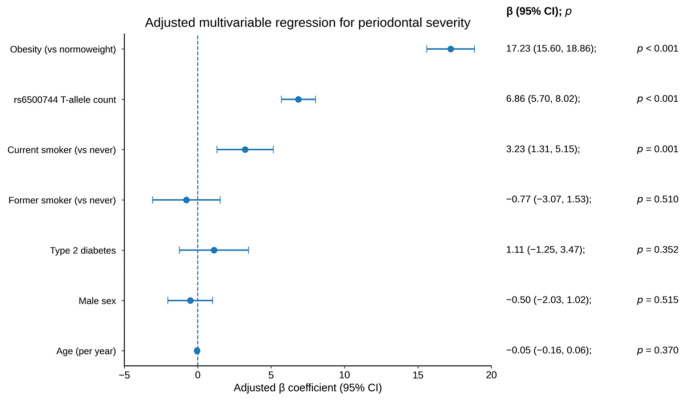
Forest plot of adjusted multivariable regression coefficients for the composite periodontal severity score in the full cohort (n = 106). Points represent adjusted β coefficients and horizontal bars represent 95% confidence intervals from the linear regression model adjusted for age, sex, obesity status, rs6500744 T-allele count, smoking status, and clinically confirmed type 2 diabetes. The vertical dashed line indicates no association (β = 0).

Adjusted Multivariable Regression Modeling. To evaluate independent predictors of periodontal breakdown, an adjusted linear regression model was constructed using the composite periodontal severity score as the dependent variable. The model included age, sex, obesity status, rs6500744 T-allele count, smoking status (current and former smoking, with never smoking as reference), and clinically confirmed type 2 diabetes. Obesity status was strongly associated with higher periodontal severity (β = 17.23, 95% CI 15.60–18.86, *p* = 5.01 × 10^−38^). The rs6500744 T-allele count was also associated with a 6.86-point increase in the severity score for each additional T allele (95% CI 5.70–8.02, *p* = 2.19 × 10^−20^). Current smoking was independently associated with higher severity (β = 3.23, 95% CI 1.31–5.15, *p* = 0.0012), whereas former smoking, age, sex, and type 2 diabetes were not statistically significant. Overall, the model explained 90.4% of the variance in periodontal disease severity (R^2^ = 0.904; adjusted R^2^ = 0.897). Multicollinearity was not detected: variance inflation factors ranged from 1.09 to 1.28, with a maximum VIF of 1.28 for current smoking. Residual diagnostics did not indicate major model-assumption violations: residual normality was acceptable (Shapiro–Wilk *p* = 0.574; Jarque–Bera *p* = 0.565), the Breusch–Pagan test did not suggest heteroscedasticity (*p* = 0.265), no externally studentized residual exceeded |3|, and no observation had Cook’s distance > 1 (maximum Cook’s distance = 0.114). Seven observations exceeded the conservative Cook’s distance threshold of 4/n, but exclusion checks did not materially change the direction or statistical significance of obesity or T-allele coefficients. Because the biological hypothesis could imply synergy between obesity and genotype, an exploratory obesity × T-allele interaction term was added to the adjusted model; this interaction was not statistically significant (β = 0.31, 95% CI −1.99 to 2.61, *p* = 0.792). Figure 5 summarizes the adjusted effect estimates for all model covariates.

Smoking-Stratified Sensitivity Analyses. To address the imbalance in smoking status, we repeated the adjusted model in smoking-restricted strata. Because CC/CT/TT genotype categories became sparse after smoking stratification, genotype–periodontitis sensitivity analyses used additive rs6500744 T-allele count rather than separate genotype categories. In non-current smokers (never and former smokers, n = 80), obesity (β = 16.80, 95% CI 14.95–18.65, *p* = 1.09 × 10^−28^) and T-allele count (β = 7.28, 95% CI 5.88–8.68, *p* = 5.19 × 10^−16^) remained associated with severity. In never-smokers only (n = 65), the corresponding estimates were β = 16.53 (95% CI 14.46–18.60, *p* = 4.24 × 10^−23^) for obesity and β = 7.16 (95% CI 5.61–8.72, *p* = 5.11 × 10^−13^) for T-allele count. In ever-smokers (former and current smokers, n = 41; additionally adjusted for pack-years), the estimates were β = 17.42 (95% CI 14.22–20.61, *p* = 7.87 × 10^−13^) for obesity and β = 6.01 (95% CI 3.97–8.05, *p* = 8.85 × 10^−7^) for T-allele count. A parsimonious current-smoker-only model (n = 26) yielded directionally consistent estimates for obesity (β = 18.44, 95% CI 14.92–21.96, *p* = 4.19 × 10^−10^) and T-allele count (β = 6.15, 95% CI 4.02–8.29, *p* = 6.06 × 10^−6^). Across sensitivity models, maximum VIFs ranged from 1.09 to 1.36. These sensitivity analyses support the robustness of the primary associations, but the current-smoker and genotype-stratified subgroups remained small; therefore, residual smoking confounding cannot be excluded.

## 3. Discussion

The present study shows that adults with obesity exhibited a more severe periodontal phenotype than normoweight controls. This phenotype was characterized by fewer remaining teeth, greater tooth loss attributed to periodontitis, elevated probing depths and clinical attachment loss, more extensive radiographic bone loss, and a higher prevalence of Stage III/IV and Grade C periodontitis. Concurrently, the *RBFOX1* rs6500744 T allele was overrepresented in the obese cohort, and periodontal severity worsened across genotypes in an allele–dose pattern (CC to CT to TT). After adjustment for age, sex, smoking status, and clinically confirmed type 2 diabetes, obesity status and T-allele burden remained associated with periodontal severity. Low VIFs, acceptable residual diagnostics, a non-significant obesity × genotype interaction, and smoking-stratified sensitivity analyses supported the internal consistency of these associations. These findings should nevertheless be interpreted as exploratory associations, not evidence of causal directionality or clinical prognostic utility.

Contextualization within Current Literature. These clinical observations are consistent with the wider epidemiological consensus linking excess adiposity to advanced periodontal breakdown. Notably, significant baseline differences in smoking status were present between the groups. Despite conducting additional smoking-stratified and current-smoker exclusion analyses, the substantial baseline imbalance in smoking status and pack-year exposure between groups represents a critical limitation. Smoking frequency matching was not implemented because participants were recruited consecutively and strict matching would have reduced recruitment feasibility and subgroup size. A propensity-score approach was considered but was not applied because the modest sample and limited overlap in smoking distributions could have produced unstable matches or weights. Therefore, smoking-restricted sensitivity analyses were used as a more transparent robustness check. Residual confounding remains plausible because the smoking strata were small, and adjustment for smoking status in the overall model may not fully capture the nuanced effects of this powerful confounder. Therefore, the independent effect attributed to obesity or genotype should be interpreted with caution. Recent systematic reviews and meta-analyses confirm that individuals with obesity have significantly higher odds of developing periodontitis, and population-based NHANES data (2011–2014) indicate that both BMI and waist circumference are independently associated with periodontal disease [9,10,23,24,25]. The larger absolute between-group differences observed in this cohort may reflect the case–control design, the targeted inclusion of participants with established obesity, the exclusion of the overweight BMI category, and the use of a comprehensive clinical–radiographic periodontal phenotype rather than a simple dichotomous disease definition. Because participants with BMI 25.0–29.9 kg/m^2^ were not included, this study cannot characterize a full adiposity dose–response relationship across normoweight, overweight, and obese categories.

Biologically, the severe tissue destruction observed in the obese cohort can be considered within a multimorbidity framework rather than interpreted as evidence of a single causal pathway. Current literature conceptualizes obesity and periodontitis as linked conditions influenced by chronic low-grade systemic inflammation, oxidative stress, insulin resistance, immune dysregulation, altered adipokine signaling, and host–microbiome dysbiosis [5,11,12,26,27,28]. These mechanisms are plausible contributors to the heightened bleeding scores, attachment loss, and alveolar bone resorption observed in the obese group, but they were not directly measured in this study.

Furthermore, the metabolic profile of the obese participants, defined by significantly elevated waist circumference, higher HbA1c values, and a greater prevalence of clinically confirmed type 2 diabetes, suggests that the advanced periodontitis developed against a backdrop of systemic metabolic dysfunction rather than existing as an isolated oral infection. Although HbA1c was recorded at baseline, the cross-sectional case–control design cannot establish longitudinal glycemic exposure or temporal sequencing between metabolic dysfunction and periodontal deterioration. The bidirectional relationship between diabetes and periodontitis is well established: hyperglycemia exacerbates periodontal inflammation, while severe periodontitis may impair glycemic control [11,29,30,31]. Importantly, our multivariable analysis showed that obesity remained associated with periodontal severity even after adjustment for diabetes, indicating that diagnosed diabetes alone does not fully account for the periodontal burden associated with obesity in this population.

Genetic Findings: The *RBFOX1* Locus. Beyond systemic metabolic mechanisms, our findings support considering the *RBFOX1* (formerly A2BP1) locus as an exploratory candidate for shared oral metabolic susceptibility. The results should not be interpreted as mechanistic proof. The exact functional role of rs6500744 in periodontal tissues remains unknown, and the present study did not measure *RBFOX1* expression, inflammatory cytokines, microbiome composition, or functional cellular responses. Previous human genetic studies have linked *RBFOX1* to obesity-related phenotypes, and experimental models suggest that Rbfox family proteins play a role in pancreatic beta-cell function and survival [19,20,22]. Furthermore, a candidate-gene study previously reported *RBFOX1* involvement in the interaction between periodontitis and metabolic traits [21], while recent biomarker reviews emphasize the need for replication before genetic markers are translated clinically [32,33]. In this context, the overrepresentation of the rs6500744 T allele among obese participants, coupled with an allele–dose gradient in periodontal destruction, raises the hypothesis that this locus may be related to adiposity-associated periodontal vulnerability, but it does not establish rs6500744 as a clinically useful biomarker.

However, these genetic findings must be interpreted with substantial caution. The absence of ancestry-informative markers or principal component adjustment is a major limitation in a genetic association study, as population stratification can create spurious genotype–disease associations that reflect underlying population structure rather than true biological causation. This significantly impacts on the interpretability of our genetic findings. Solid validation, including replication in larger, ethnically diverse cohorts with ancestry control, is often lacking in genetic biomarker studies, which are subject to methodological heterogeneity and inconsistent replication across populations [14,17,33]. Therefore, the association observed in this study should be considered hypothesis-generating. Stringent validation, including replication in larger multicenter and ethnically diverse cohorts, ancestry control, haplotype analysis, microbiome/immune phenotyping, and functional assays, is required before rs6500744 can be established as a periodontal risk marker.

Clinical Implications. From a clinical perspective, these results support adopting an integrated model for risk assessment and disease management in patients with obesity. In addition to standard periodontal therapies, screening for smoking exposure, diabetes status, and other metabolic traits may support closer collaboration between dental and medical professionals [11,30]. This cross-disciplinary approach is particularly important given evidence that patients with obesity may experience a poorer or delayed response to non-surgical periodontal therapy, frequently requiring more intensive treatment [32,34,35]. However, the present study results are not sufficient to develop or deploy a prognostic model for periodontitis. A clinically useful model would require prospective data, predefined predictors, robust handling of smoking, diabetes, and adiposity, assessment of discrimination and calibration, decision curve analysis, and external validation. Until such validation is available, *RBFOX1* rs6500744 should be regarded as a research marker only.

Strengths and Limitations. This study possesses several notable strengths, including balanced group sizes, comprehensive clinical and radiographic periodontal phenotyping, the application of multiple genetic models, and robust adjusted multivariable analysis.

However, several limitations should be acknowledged. The observational case–control design precludes assertions of causality or temporal sequencing; therefore, the present data cannot establish whether obesity preceded periodontitis, whether periodontal inflammation influenced metabolic status, or whether *RBFOX1* rs6500744 affects one or both conditions [36]. Additionally, the study population was recruited from a single Romanian center and associated practices, limiting generalizability across ethnic, geographic, and socioeconomic settings. Participants with BMI 25.0–29.9 kg/m^2^ were excluded by design, which sharpened the contrast between normoweight and obese groups but limits generalizability and prevents evaluation of a graded adiposity–periodontitis dose–response relationship. Smoking differed between groups; although the smoking-stratified and smoker-exclusion sensitivity analyses yielded directionally consistent estimates, residual confounding remains possible because strata were small and pack-year exposure remained uneven. The sample size is also small for a genetic association study, particularly because only 14 TT homozygotes were included, which limits the precision and reproducibility of genotype-stratified estimates. The composite periodontal severity score was study-specific and not externally validated; although it did not include obesity status, genotype, age, sex, smoking, diabetes, BMI, waist circumference, pack-years, or HbA1c, its high R^2^ should be interpreted in the context of a composite periodontal burden outcome, not as evidence of a validated prognostic model. Multiple secondary and genotype-stratified comparisons were performed without formal multiplicity correction; therefore, borderline or subgroup findings should be interpreted as exploratory and hypothesis-generating. Furthermore, the study evaluated only a single polymorphism and did not include ancestry-informative markers, principal component analysis, longitudinal HbA1c trajectories, inflammatory biomarker quantification, microbiome profiling, *RBFOX1* expression assessment, or functional assays, all of which are necessary to elucidate underlying biological mechanisms.

## 4. Materials and Methods

Study Design and Population. This case–control study enrolled 106 adults, recruited consecutively from the 2nd Department of Surgery, Emergency County Clinical Hospital of Targu Mures, Romania, and associated private practices from October 2022 to December 2024. The cohort was divided into two groups: an obese group of 53 individuals with obesity (BMI ≥ 30 kg/m^2^) and a normoweight control group of 53 individuals with a BMI between 18.5 and 24.9 kg/m^2^. Participants with BMI 25.0–29.9 kg/m^2^ were excluded a priori to create a clear contrast between normoweight and obese groups for this exploratory case–control comparison. This design choice improves group separation but limits generalizability and prevents assessment of a continuous adiposity dose–response relationship. Waist circumference and BMI were both measured to capture central and overall adiposity. Baseline demographic and clinical data were collected for all participants, including age, sex, smoking status and pack-years, HbA1c, and clinically confirmed history of type 2 diabetes based on medical records or ongoing antidiabetic therapy. Only baseline data were used for this analysis.

A priori sample size calculation was performed for the primary outcome: the association between the *RBFOX1* rs6500744 polymorphism and obesity group status. Based on previous literature investigating metabolic-associated genetic variants, we anticipated an exposure rate of the target allele (carriers of at least one T allele) of approximately 30% in the normoweight control group. To detect an odds ratio (OR) of 2.5 under a dominant genetic model (CT + TT vs. CC) with 5% Types I error rate (α = 0.05) and 80% power (1 − β = 0.80) in a two-tailed test, the required sample size was calculated to be approximately 49 participants per group. To account for potential genotyping failures or incomplete clinical records, we recruited slightly above this threshold, enrolling 53 participants per group (n = 106 total).

Although adequate for the prespecified group comparison, this sample size remains modest for genetic association and multivariable genotype-stratified analyses. Consequently, genotype-specific estimates, particularly for the TT homozygote subgroup, were interpreted as exploratory.

Eligibility Criteria. Eligible participants were at least 18 years old, had sufficient teeth for a valid periodontal assessment, and had complete clinical and genetic data. Exclusion criteria comprised partial records, oral issues preventing accurate probing, or missing rs6500744 genotyping. Standard study exclusions applied: recent periodontal treatment, recent systemic antibiotics, pregnancy, or severe general conditions affecting inflammation.

Clinical and Radiographic Periodontal Assessment. Prior to the commencement of the study, all clinical periodontal examinations were performed by two calibrated periodontists. To minimize bias, the examiners were strictly blinded to the participants’ obesity status, systemic medical history, and subsequent genetic data. Calibration was conducted prior to the study on a subset of 8 non-study patients exhibiting varying stages of periodontal disease. Inter-examiner reliability for probing pocket depth (PPD) and clinical attachment loss (CAL) was evaluated, demonstrating a high degree of agreement (Cohen’s kappa = 0.86 for measurements within 1 mm). Intra-examiner reliability was similarly verified (Cohen’s kappa = 0.89).

A full-mouth periodontal exam was done at baseline. Clinical parameters included mean and maximum probing pocket depth, mean and maximum clinical attachment loss, bleeding on probing, plaque index, and gingival index. The number of teeth, teeth lost to periodontitis, furcation involvement, and tooth mobility were noted. Radiographic bone loss was calculated as a percentage, with a bone loss/age ratio used to aid grading when longitudinal data were unavailable. Disease was categorized as localized or generalized based on affected teeth. A study-specific composite periodontal severity score was used as a continuous descriptive periodontal burden outcome. The score was calculated as: 5.5 × mean PPD + 6.0 × mean CAL + 0.18 × BOP (%) + 0.35 × radiographic PBL (%) + 2.5 × number of teeth lost due to periodontitis + 2.0 × maximum furcation score + 2.0 × maximum mobility score, rounded to one decimal place. Higher values indicate greater periodontal burden. The score did not include obesity status, BMI, waist circumference, rs6500744 genotype or T-allele count, age, sex, smoking status, pack-years, HbA1c, or diabetes status, and therefore did not overlap with the exposure/covariate variables used in the adjusted regression model. The score should be interpreted as a study-specific descriptive index rather than an externally validated clinical scale (Table 1).

Periodontal Diagnosis and Classification. Periodontitis diagnosis and case characterization were strictly based on the 2017 World Workshop (AAP-EFP) classification system [24]. A confirmed case required interdental CAL on more than one non-adjacent tooth after other causes of attachment loss were excluded. Disease staging (I–IV) used severity and complexity variables: CAL, radiographic bone loss, PPD, furcation involvement, and tooth loss. Grading (A–C), which reflects biological risk and progression rate, was first estimated indirectly with the radiographic bone loss/age relationship. The grade was then adjusted using modifiers: smoking exposure and diabetes status. Final case definitions combined Stage, Grade, and Extent (e.g., Stage III, Grade C, generalized) [8].

Genotyping of the *RBFOX1* rs6500744 Polymorphism. For DNA isolation, 2 mL of venous blood was collected in tubes containing ethylenediaminetetraacetic acid (EDTA) from each subject included in the study. Genomic DNA was isolated using the PureLink Genomic DNA kit (Thermo Fisher Scientific, Carlsbad, CA, USA) according to the manufacturer’s protocol. DNA quantification was performed using an Eppendorf BioSpectrometer (Eppendorf, Wien, Austria GmbH). Extracted DNA was stored at −20 °C until further analysis.

*RBFOX1* rs6500744 genotyping was performed using TaqMan^®^ SNP Genotyping Assay (C_2943260_10; Thermo Fisher Scientific, Carlsbad, CA, USA) and 7500 Fast Dx Real-Time PCR system (Applied Biosystems, Foster City, CA, USA).

Each participant was classified as CC, CT, or TT. For analysis, genetic data were modeled in three ways: allelic model (number of T alleles: 0, 1, or 2), dominant model (T-allele carriers [CT + TT] vs. CC), and recessive model (TT vs. [CC + CT]). For descriptive purposes, CC was treated as the reference category, CT as the heterozygous carrier category, and TT as the homozygous T-allele category; these categories should not be interpreted as clinically validated risk strata.

All laboratory personnel responsible for DNA isolation, quantification, and TaqMan^®^ SNP genotyping were entirely blinded to the participants’ clinical periodontal phenotype and obesity group allocation.

Study Outcomes. The primary outcome of the study was the association between the rs6500744 polymorphism and group status (obesity test group versus normoweight control group). Secondary outcomes investigated the relationship between the genotype and both continuous baseline periodontal severity metrics and the categorical 2017 periodontal classifications. The distribution of the dominant and recessive genetic models across the two groups was also analyzed.

Statistical Analysis. Statistical analyses were performed on the individual-level dataset. Following normality testing, continuous variables were summarized as means ± standard deviations (SD) or as medians with interquartile ranges, while categorical variables were expressed as absolute frequencies and percentages. Between-group comparisons were performed using the independent-samples *t*-test for normally distributed continuous data, or the Mann–Whitney U test for non-parametric data. Categorical variables, including genotype distributions, were compared using the chi-square test or Fisher’s exact test where appropriate. Allele frequencies were calculated via direct counting. The strength of the associations between genotype/allele distributions and group membership was quantified using odds ratios (ORs) with 95% confidence intervals (CIs) under the defined genetic models. Departures from Hardy–Weinberg equilibrium were assessed using chi-square goodness-of-fit testing based on observed and expected genotype counts. For the case–control genetic analysis, the control-group HWE test was treated as the primary genotyping quality-control assessment; HWE testing in the overall cohort and obese subgroup was reported descriptively only. Multivariable linear regression used the composite periodontal severity score as the dependent variable; independent variables included obesity status, rs6500744 T-allele count, age, sex, current and former smoking, and clinically confirmed type 2 diabetes. An exploratory obesity × T-allele interaction term was also tested. Model diagnostics included variance inflation factors, standardized and externally studentized residuals, residual-versus-fitted and Q-Q plots, Shapiro–Wilk and Jarque–Bera residual normality tests, Breusch-Pagan testing for heteroscedasticity, leverage, and Cook’s distance. Sensitivity analyses were conducted by smoking status, including non-current smokers, never-smokers only, ever-smokers with additional adjustment for pack-years, and a parsimonious current-smoker-only model because of limited stratum size. Because multiple clinical, genetic-model, and genotype-stratified comparisons were performed, the analyses were interpreted as exploratory; no formal multiplicity correction was applied, and borderline or subgroup findings were interpreted cautiously. A two-sided *p*-value < 0.05 was considered statistically significant for the primary adjusted models. Exact *p*-values are reported whenever possible, including in scientific notation for values below 0.001. Given the modest sample size, analyses were not intended to generate a validated prognostic model. A prognostic model would require individual-level longitudinal data, model calibration and discrimination assessment, and external validation.

Ethical Issues. The study was conducted in strict accordance with the Declaration of Helsinki and local administrative guidelines. The protocol was reviewed and approved by the Ethics Committee of the George Emil Palade University of Medicine, Pharmacy, Science, and Technology of Targu Mures, Romania (Approval No. 1863/15.09.2022). All participants provided written informed consent prior to clinical examination and sample collection. The STROBE Statement checklist was followed to ensure transparent and comprehensive reporting of this observational study (Supplementary Materials).

## 5. Conclusions

In this exploratory case–control cohort, obesity was associated with a more severe periodontal phenotype. The *RBFOX1* rs6500744 T allele was more prevalent in participants with obesity and showed an exploratory allele–dose association with periodontal severity. After adjustment for age, sex, smoking status, and clinically confirmed type 2 diabetes, obesity and T-allele count remained associated with severity; these associations were directionally consistent in smoking-stratified sensitivity analyses, while the obesity × T-allele interaction was not statistically significant. However, the observational design, small genotype subgroups, smoking imbalance, multiple testing, exclusion of overweight participants, single-center recruitment, and absence of ancestry, biomarker, microbiome, or functional data preclude causal or prognostic claims. The rs6500744 polymorphism should therefore be considered a hypothesis-generating research marker rather than a validated biomarker. Larger multicenter, ancestry-controlled, prospective studies across the full BMI spectrum are needed to determine whether this locus contributes to obesity-associated periodontitis and whether it can improve clinically useful risk prediction.

## Figures and Tables

**Table 1:** Components and weighting of the composite periodontal severity score.

Component	Measurement	Weight/Scaling	Overlap with Regression Covariates
Pocket depth	Mean probing pocket depth (PPD, mm)	5.5 × mean PPD	No
Attachment loss	Mean clinical attachment loss (CAL, mm)	6.0 × mean CAL	No
Inflammation	Bleeding on probing (BOP, %)	0.18 × BOP (%)	No
Radiographic destruction	Radiographic periodontal bone loss (PBL, %)	0.35 × PBL (%)	No
Tooth loss	Number of teeth lost due to periodontitis	2.5 × tooth-loss count	No
Complexity	Maximum furcation involvement score	2.0 × maximum furcation score	No
Complexity	Maximum tooth mobility score	2.0 × maximum mobility score	No
Variables excluded from the score	BMI/obesity status, waist circumference, rs6500744 genotype or T-allele count, age, sex, smoking status, pack-years, HbA1c, and diabetes status	0; not used in the score	Some listed variables were exposures/covariates, but none were included in the score

## Data Availability

The original contributions presented in the study are included in the article, further inquiries can be directed at the corresponding author.

## Supplementary Materials

The following supporting information can be downloaded at: https://www.mdpi.com/article/10.3390/ijms27114867/s1.

## Abbreviations

The following abbreviations are used in this manuscript:

A2BP1	Former designation for the *RBFOX1* gene
AAP-EFP	American Academy of Periodontology and European Federation of Periodontology (referencing the 2017 World Workshop classification system)
BMI	Body mass index
BOP	Bleeding on probing
CAL	Clinical attachment loss
CI	Confidence interval
GWAS	Genome-wide association studies
HbA1c	Glycated haemoglobin
NHANES	National Health and Nutrition Examination Survey (referencing 2011–2014 population-based data)
OR	Odds ratio
PBL	Periodontal bone loss
PCR	Polymerase chain reaction
PPD	Probing pocket depth
SD	Standard deviations
WC	Waist circumference
