# Supplementary material for: Association of the RBFOX1 rs6500744 Polymorphism with Periodontal Disease Severity in Adults with Obesity: A Case–Control Study
Source: Int J Mol Sci. 2026 May 28;27(11):4867. doi: 10.3390/ijms27114867 (PMC13257284; doi:10.3390/ijms27114867)
Supplement: Supplementary file 1 [file ijms-27-04867-s001.zip › ijms-4304422-supplementary.pdf]

**SUPPLEMENTARY MATERIALS**

**S1. STROBE Statement Checklist**

| Item No | Recommendation | Page/Section |
|---|---|---|
| **Title and Abstract** | Indicate the study's design with a commonly used term in the title or the abstract<br>Provide an informative and balanced summary of what was done and found | Title, Abstract |
| **Background** | Explain the scientific background and rationale for the investigation<br>being reported | Introduction |
| **Objectives** | State specific objectives, including any prespecified hypotheses | Introduction |
| **Study Design** | Present key elements of study design early in the paper | Methods/Study Design |
| **Setting** | Describe the setting, locations, and relevant dates, including periods of recruitment, exposure, follow-up, and data collection | Methods/Study Design |
| **Participants** | Give the eligibility criteria, and the sources and methods of case<br>ascertainment and control selection. Give the rationale for the choice of cases and controls | Methods /Eligibility |
| **Variables** | Clearly define all outcomes, exposures, predictors, potential confounders, and effect modifiers | Methods/Clinical Assess |
| **Data sources/ measurement** | For each variable of interest, give sources of data and details of<br>methods of assessment (measurement). Describe comparability of assessment methods | Methods |
| **Bias** | Describe any efforts to address potential sources of bias | Methods / Ext. exclusions |
| **Study size** | Explain how the study size was arrived at | Methods |
| **Statistical methods** | Describe all statistical methods, including those used to control confounding | Methods / Statistical Analysis |
| **Main results** | Give unadjusted estimates and, if applicable, confounder-adjusted estimates and their precision (eg, 95% confidence interval) | Results |
| **Limitations** | Discuss limitations of the study,taking into account of potential bias or imprecision | Discussion/Limitations |
